# Exploiting the Anti-Inflammatory Potential of White *Capsicum* Extract by the Nanoformulation in Phospholipid Vesicles

**DOI:** 10.3390/antiox10111683

**Published:** 2021-10-25

**Authors:** Ilaria Pappalardo, Anna Santarsiero, Maria De Luca, Maria Assunta Acquavia, Simona Todisco, Carla Caddeo, Giuliana Bianco, Vittoria Infantino, Giuseppe Martelli, Antonio Vassallo

**Affiliations:** 1 Department of Science, University of Basilicata, Viale dell’Ateneo Lucano 10, 85100 Potenza, Italy; ilaria.pappalardo@unibas.it (I.P.); anna.santarsiero@unibas.it (A.S.); maria.deluca@unibas.it (M.D.L.); maria.acquavia@unibas.it (M.A.A.); simona.todisco@unibas.it (S.T.); giuliana.bianco@unibas.it (G.B.); vittoria.infantino@unibas.it (V.I.); giuseppe.martelli@unibas.it (G.M.); antonio.vassallo@unibas.it (A.V.); 2 ALMACABIO Srl, C/so Italia 27, 39100 Bolzano, Italy; 3 KAMABIO Srl, Via Al Boschetto 4/B, 39100 Bolzano, Italy; 4 Thema Informatik Srl, Via Ressel 2/F, 39100 Bolzano, Italy; 5 Department of Scienze della Vita e dell’Ambiente, Sezione di Scienze del Farmaco, University of Cagliari, Via Ospedale 72, 09124 Cagliari, Italy; 6 Spinoff TNcKILLERS s.r.l., Viale dell’Ateneo Lucano 10, 85100 Potenza, Italy

**Keywords:** white *Capsicum* extract, phospholipid vesicles, anti-inflammatory, ROS, NO, cells

## Abstract

The peppers of the *Capsicum* species are exploited in many fields, as flavoring agents in food industry, or as decorative and therapeutic plants. Peppers show a diversified phytochemical content responsible for different biological activities. Synergic activity exerted by high levels of antioxidant compounds is responsible for their important anti-inflammatory property. A methanolic extract was obtained from a new pepper genotype and tested for anti-inflammatory activity. The extract was incorporated into phospholipid vesicles to increase the bioavailability of its bioactive components. Two types of phospholipid vesicles were produced, conventional liposomes and Penetration Enhancer containing Vesicles (PEVs). They were tested in human monoblastic leukemia U937 cell line, showing no cytotoxic effect. The intracellular reactive oxygen species (ROS) and nitric oxide (NO) levels were measured to value the in vitro efficacy of the vesicles in regulating inflammatory responses. Liposomal incorporation significantly reduced ROS levels in extract-treated LPS-activated cells. Furthermore, LC-MS/MS analyses demonstrated that liposomes facilitated the transport of the extract components across the cell membrane and their accumulation into the cytoplasm.

## 1. Introduction

Plants are an important source of chemical compounds mainly derived from their secondary metabolites with valuable properties to consumers [1].

Peppers (*Capsicum* spp.) are among the oldest cultivated crops in warm climate regions worldwide and are a fundamental reservoir of bioactive compounds that contribute to the taste, color, and flavor of the fruits. *Capsicum* species are annual herbaceous plants belonging to the Solanaceae family, including domesticated species such as *C.* *baccatum*, *C.* *annuum*, *C.* *pubescen*, *C.* *frutescens*, and *C.* *chinense*, which display a wide range of fruit size, shape, color, and pungency [2].

Common peppers are mainly used in the food sector, as flavoring agents, for example, but they are also cultivated as decorative and therapeutic plants. Peppers show different biological activities due to their high and diverse phytochemical content, which includes capsaicinoids, carotenoids, phenolic compounds, vitamins, and minerals, and whose expression profile depends on the fruit part, the genotype, the ripening stage, the climatic conditions, the processing and storage practices [3,4]. Capsaicinoids are a group of alkaloids produced in variable amounts in hot peppers and their chemical structure influences the pungency of *Capsicum* species. They are probably produced as deterrents against certain herbivores and fungi, and major representatives are capsaicin and dihydrocapsaicin. Capsaicinoids have a rather strong biological activity and a potential pharmacological and clinical application for the treatment of neurological and musculoskeletal pain, and inflammatory and oxidative diseases [5,6,7,8,9,10,11,12,13,14].

Recently, capsinoids have been found in some varieties of peppers. They are non-pungent compounds with a similar structure to capsaicinoids. Their mechanisms of action are poorly understood, but they have shown some interesting properties, such as antimicrobial activity [4].

Peppers are also good sources of vitamins, showing high levels of vitamin C, E, provitamin A, and folate, and they are also rich in minerals, including iron, calcium, and manganese [15]. Mostly, *Capsicum* fruits contain high levels of antioxidants primarily responsible for the antiseptic, antimetastatic, antifungal, antiviral, anti-inflammatory, and immunomodulatory properties of peppers [3,4]. The radical scavenging activity of peppers is also influenced by the synergism between the total antioxidants. Peppers are generally rich in different types of carotenoids, such as β-carotene, capsanthin, violaxanthin, lutein, zeaxanthin, capsorubin, antheraxanthin, and others [3,16,17]. The excellent antioxidant properties of these compounds, which protect cells from free radicals by scavenging reactive oxygen species (ROS), are due to the presence of a conjugated double bond system in their chemical structure [4]. Peppers are also rich in phenolic compounds, mostly flavonoids and phenolic acid derivatives that have health-promoting effects, as they protect the body from the damage produced by oxidative agents [18,19]. Among phenolics, quercetin is one of the most potent compounds. It is a flavonol with anticancer, antiviral, antiprotozoal, anti-hypertensive, anti-inflammatory and antimicrobial effects [20], also employed for the treatment of eye and cardiovascular diseases, arthritis, allergic, metabolic, and inflammatory disorders [21,22,23,24].

Inflammation is a defence mechanism of the body against detrimental stimuli including pathogens (bacteria, viruses, and parasites) and/or toxins released by pathogens, trauma, heat, or damaged cells [25]. The immune system eliminates the harmful agents responsible for tissue damage and initiates the healing process to restore homeostasis. The unconstrained acute inflammatory response could evolve in chronic inflammation, which may increase the risk for different chronic diseases, such as diabetes, cardiovascular diseases, cancer, arthritis, and joint diseases [26]. Immune cells resident in the damaged tissues, mostly resident macrophages and dendritic cells, recognize the noxious agents thanks to pattern recognition receptors on their cell surface. Subsequently, inflammatory signaling pathways (i.e., NF-κB signaling cascade) are activated with consequent changes in vascular permeability, leukocytes chemotaxis to the site of damage, and the release of inflammatory mediators [27].

Under inflammatory conditions, triggered immune cells undergo metabolic shifts to fulfil the new cellular demands of energy and biomolecules [28,29]. Metabolites produced could act as signaling molecules or be used for the synthesis of inflammatory mediators. In lipopolysaccharide (LPS)-triggered macrophages, as a consequence of the rewired Krebs cycle, there is an accumulation of different metabolites among which is citrate [30]. Citrate represents a key signal for the activation and functions of immune cells and has a central role in the inflammatory cascade [31]. In fact, citrate contributes to the synthesis of chemical mediators of inflammation, namely ROS, nitric oxide (NO), and prostaglandin E_2_ (PGE_2_), and can affect cytokine secretion [32,33,34]. The inhibition of the citrate pathway, composed of citrate, mitochondrial citrate carrier and ATP citrate lyase (ACLY), by synthetic [35,36] and natural compounds, such as hydroxycitric acid (HCA) [18,34,37], counteracts inflammation and oxidative stress.

Non-steroidal anti-inflammatory drugs (NSAIDs) are commonly used to control inflammation by inhibition of PGE_2_ biosynthesis, but they can cause serious side effects. There is ongoing research for new anti-inflammatory compounds that can be associated with or can replace NSAIDs and can act on other targets of the inflammatory cascade. Plant secondary metabolites have attracted the interest of several researchers over the last few years for their numerous biological activities including anti-inflammatory properties, especially phenolic compounds and quercetin. Unfortunately, they are generally poorly soluble and bioavailable, and chemically unstable. The incorporation of bioactive compounds or extracts from plants into nanocarriers has been shown to protect from degradation, and to increase solubility and bioavailability [38,39,40]. For example, the nanoencapsulation of ACLY inhibitor HCA into liposomes enhanced its anti-inflammatory activity by increasing its intracellular uptake [41]. Nanocarriers, such as phospholipid vesicles, represent a huge amount of promise for drug delivery, since they allow the successful treatment of diseases with minimal side effects. They are nanosized particles with elevated surface area to volume ratio. Thanks to their versatile and tunable composition and structure, phospholipid vesicles improve pharmacokinetics, pharmacodynamics, solubility and stability of drugs, decrease their toxicity, and guarantee site-specific delivery [42,43]. Intensive research has been devoted to the development of new classes of phospholipid vesicles that are capable of enhancing the efficacy of the payload in the skin, such as transfersomes, ethosomes and PEVs (Penetration Enhancer containing Vesicles) [44].

In a previous work [45], we conducted a comprehensive phytochemical profiling of a methanolic extract of a new pepper genotype deriving from an original crossing combination between Habanero white, from *C. chinense*, and *C. annuum*. Furthermore, we demonstrated the anti-inflammatory properties of the extract, as it was able to reduce ROS levels in LPS-activated cells. In the present study, we investigated the anti-inflammatory activity of this white *Capsicum* extract incorporated into phospholipid vesicles to evaluate a potential enhancement of its biological activity.

## 2. Materials and Methods

### 2.1. Materials

Phospholipon90G (>90% phosphatidylcholine; P90G) was purchased from Lipoid GmbH (Ludwigshafen, Germany). Propylene glycol was purchased from Galeno (Carmignano, Prato, Italy). LC-MS grade acetonitrile was obtained from Sigma-Aldrich (Milan, Italy). Formic acid (99%), used as additive of aqueous mobile phase, was purchased from Carlo Erba Srl (Milan, Italy). Deionized water was obtained with a Milli-Q RG system (Millipore, Bedford, MA, USA).

L-glutamine, penicillin/streptomycin solution, lipopolysaccharide from *Salmonella enterica* serotype typhimurium (LPS), fetal bovine serum (FBS), Roswell Park Memorial Institute 1640 (RPMI 1640), and phorbol 12-myristate 13-acetate (PMA) were purchased from Sigma-Aldrich (St. Louis, MO, USA). Furthermore, 6-Carboxy-2′,7′-Dichlorodihydrofluorescein Diacetate (DCF-DA) and 4-amino-5-methylamino-2′,7′-difluorofluorescein diacetate (DAF-FM Diacetate) were obtained from Thermo Fisher Scientific (San Jose, CA, USA).

### 2.2. Extract Preparation

In a previous work [45], we reported the preparation of an extract from a pepper species obtained from a specific breeding program. The white *Capsicum* extract was prepared by optimizing the protocol of Wahyuni et al. [46]: 3 mL of methanol was added to 0.5 g of lyophilized peppers; the sample was sonicated for 15 min (25 °C) in a Sonorex Super RK 100/H sonicator (Bandelin electronic, Berlin, Germany); and the methanolic extract was filtered through 0.20 µm nylon filters, which are recommended for methanolic matrices as they do not procure changes in flow rate or bubble point of the membrane, nor visible chemical attack. The extract was subjected to solvent evaporation (Laborota 4000 efficient, Heidolph, Schwabach, Germany).

### 2.3. Vesicle Preparation and Characterization

For the preparation of conventional liposomes, P90G and white *Capsicum* extract were weighed in a glass vial and dispersed in water (Table 1). The dispersion was sonicated (10 cycles 5 s on/2 s off + 10 cycles 3 s on/2 s off; 13 microns of probe amplitude) with a Soniprep 150 (MSE Crowley, London, UK). For the preparation of Penetration Enhancer containing Vesicles (PEVs), propylene glycol (PG) was dispersed in water along with P90G and the extract (Table 1). Thereafter, the dispersion was sonicated as reported above for the preparation of liposomes.

For comparative purposes, empty vesicles and empty PG-PEVs were prepared following the above procedure, but without the addition of white *Capsicum* extract (Table 1).

The average diameter, polydispersity index (PI, a measure of the width of size distribution), and zeta potential of the vesicles were determined via dynamic and electrophoretic light scattering using a Zetasizer nano-ZS (Malvern Panalytical, Worcestershire, UK). Samples (*n* > 10) were diluted with water (1:100) and analyzed at 25 °C.

The stability of the formulations was evaluated by monitoring vesicle mean size, PI, and zeta potential over three months at 4 ± 2 °C.

The vesicle dispersions were purified from the non-incorporated active compounds by dialysis. Each sample (1 mL) was loaded into Spectra/Por^®^ tubing (12–14 kDa MW cut-off; Spectrum Laboratories Inc., DG Breda, The Netherlands), previously rinsed in water, and dialyzed against water (2 L) for 2 h to allow the removal of the non-incorporated components of the extract. Unpurified and purified vesicles were disrupted with methanol (1:100) [47,48] and dried under N_2_ flow. The solid residues were redissolved in 80 μL of MeOH and filtered through 0.20 μm nylon filters before injection in a liquid chromatography coupled to a tandem mass spectrometry (LC-MS/MS) system (see Section 2.7). The content of capsaicin was determined, and the entrapment efficiency (E) was calculated as the percentage of the amount of capsaicin in purified vesicles, according to the following Formula (1):

(1) $$E=\left(\frac{\mathrm{amount}\;\mathrm{of}\;\mathrm{Capsaicin}\;\mathrm{in}\;\mathrm{purified}\;\mathrm{vesicles}}{\mathrm{amount}\;\mathrm{of}\;\mathrm{Capsaicin}\;\mathrm{in}\;\mathrm{unpurified}\;\mathrm{vesicles}}\right)\;\times \;100$$

### 2.4. Cell Culture and Treatments

Human monoblastic leukemia U937 cell line (HTL94002-Interlab Cell Line Collection, IRCCS San Martino Hospital, Genoa, Italy) was maintained in RPMI 1640 medium supplemented with 10% (*v*/*v*) fetal bovine serum, 2 mM l-glutamine, 100 U/mL penicillin, and 100 µg/mL streptomycin at 37 °C in 5% CO_2_ in a water-saturated atmosphere. U937 cells were differentiated to macrophages by using 10 ng/mL PMA. U937/PMA cells were treated with 5 or 25 µg/mL free white *Capsicum* (WC) extract, WC liposomes or WC PG-PEVs (both non-dialyzed), and, where appropriate, stimulated with 200 ng/mL LPS. The vesicle formulations were sterilized by using 0.22 µm syringe filters (STARLAB, Milan, Italy).

### 2.5. Cytotoxicity Assay

Cells were cultured into 96-well plates (5 × 10^4^ cells/well) and treated with free WC, WC liposomes or WC PG-PEVs (both non-dialyzed). The effects on the U937 cell number were determined using a Millipore Scepter™ handheld automated cell counter (Merck Millipore, Darmstadt, Germany) after 48 h of incubation, according to the manufacturer’s instructions.

### 2.6. ROS and NO Detection

To evaluate ROS and NO levels, U937 cells were seeded at the density of 1 × 10^5^ cells/well, differentiated with 10 ng/mL PMA, and triggered by 200 ng/mL of LPS in both the presence and absence of free WC, WC liposomes or WC PG-PEVs (both non-dialyzed).

After 24 h, intracellular total ROS were detected by using DCF-DA, a chemically reduced form of fluorescein used as a probe for ROS in cells. After cleavage of the acetate groups by intracellular esterases and oxidation, the non-fluorescent H_2_DCFDA is converted to highly fluorescent 2′,7′-dichlorofluorescein (DCF, Ex/Em: ~492–495/517–527 nm) [49]. At the end of treatments, the cell culture medium was removed, and DCF-DA was added to the cell pellet at the final concentration of 10 µM. After 30 min incubation at 37 °C in the dark, 100 µL of samples were transferred into a black microtiter plate in triplicate and the fluorescence was revealed using the GloMax plate reader (Promega, Madison, WI, USA).

NO concentrations were measured by using DAF-FM Diacetate, after 24 h from the beginning of treatments. DAF-FM diacetate is non-fluorescent until it reacts with NO to form a fluorescent benzotriazole (Ex/Em of DAF-FM: ∼495/515 nm) [49]. U937/PMA cells were incubated with 2.24 µM DAF-FM diacetate for 30 min in the dark. The fluorescence was measured using GloMax plate reader. Each experiment was performed in triplicate.

### 2.7. LC-MS/MS Analysis

To quantify the cellular uptake of the white *Capsicum* extract, U937/PMA cells were seeded into 6-well plates at a density of 2.5 × 10^5^ cells/well, treated with 5 µg/mL and 25 µg/mL of extract (as free or incorporated in liposomes and PG-PEVs) for 24 h, and pelleted at 1200 rpm for 5 min. Supernatants were removed. To pull out the extract, 500 µL of 40% MeOH with 0.1% (*v*/*v*) formic acid was added to the cells, which were lysed by sonication (1 min on/1 min off, 10 min total), mixed, and placed on ice for 15 min. Lysates were then centrifuged at 1500 rpm for 5 min. Supernatants were collected for LC-MS analysis. To ensure that all of the extract was pulled out from cells, another amount of 250 µL of 40% MeOH with 0.1% (*v*/*v*) formic acid was added to the cell pellets and suspended again. After 15 min on ice, the suspensions were centrifuged at 14,000 rpm for 5 min, and all supernatants were pooled together [20,41]. The supernatants were dried with vacuum-centrifugation using a Concentrator plus (Eppendorf AG, Hamburg, Germany). Before the cellular uptake quantification, samples were reconstituted with 80 µL of LC-MS grade MeOH, filtered through 0.20 µm nylon filters and 15 µL aliquots were injected in the LC-MS system. The protein concentration in each sample was determined by the Bradford assay [50], and cell lysates were checked to verify success of the lyses step and to normalize multiple samples for side-by-side comparison.

Capsaicin content, for both entrapment efficiency evaluation and intracellular uptake quantification, was assayed using an HPLC system coupled with a linear ion trap quadrupole (LTQ) mass spectrometer equipped with an electrospray ionization source, both from Thermo Fisher Scientific (Bremen, Germany). The chromatographic separation was performed on a revered-phase Luna C18 (2) column (150 × 4.6 mm internal diameter, 3.0 µm particle size, 100 Å pore size from Phenomenex Torrance, CA, USA). The mobile phase consisted of water containing 0.1% *v*/*v* of formic acid (solvent A) and acetonitrile (solvent B). The gradient elution program was adapted from previous works [45,51] as follows: from 0% B and increased to 35% B over 5 min. After holding at 35% for 5 min, mobile phase B was increased to 40% over 5 min. Then, the composition was linearly increased to 85% B in 15 min, to return to initial conditions at min 32.0. The initial conditions were held for 8 min in order to recondition the column. The sample injection volume was set at 15 µL and the flow rate was 0.8 mL min^−1^, with a 4:1 post-column split, thus allowing 200 µL/min to enter the ESI source, operating in positive ionization mode. MS analyses were performed by using product ion scan mode. Precursor ion at *m*/*z* 306.0, corresponding to the protonated ion of capsaicin [M + H]^+^, was selected for the screening of capsaicin in the samples of interest. All fragment ions, obtained by Collision Induced Dissociation (CID) of the precursor ion, were scanned in the range 80–650 *m*/*z*. Pure nitrogen (99.996%) was used as sheath gas, at a flow of 60 arbitrary units (a.u.). The following parameters were applied for MS ionization: needle voltage, +4.50 kV; capillary voltage, +13.0 V; and capillary temperature, 350 °C. An isolation window of 6 *m*/*z* and a collision energy of 5.2 eV (15%) were used for precursor ion fragmentation. All the data were exported and processed with Xcalibur 2.2 software (Thermo Fisher Scientific) [52].

### 2.8. Statistical Analysis of Data

Statistical significance of differences was determined by using one-way analysis of variance (ANOVA) followed by Dunnett’s test or Tukey’s post hoc test for multiple comparisons. For LC-MS analysis, Student’s *t*-test was used. Results are shown as mean ± standard deviation (S.D.) of three independent experiments. The statistical method employed is detailed in each figure legend. Asterisks indicate the significance: * *p* < 0.05, ** *p* < 0.01, and *** *p* < 0.001. When Tukey’s test was performed, different letters in figures denote significant differences between treatments at *p* < 0.05.

## 3. Results

### 3.1. Vesicle Design and Characterization

The present study aimed to develop a vesicular formulation for the delivery of white *Capsicum* extract to the skin. Two types of phospholipid vesicles, liposomes, and PEVs, were produced to enhance the bioavailability of the extract components through nanoincorporation and facilitate the interaction with cells to achieve an increased intracellular accumulation and consequent biological response.

In order to discriminate between the activity of the white *Capsicum* extract and the effect of the nanocarriers, WC-loaded liposomes and PG-PEVs were prepared, characterized, and compared with empty liposomes, empty PG-PEVs, and free WC extract.

Light scattering results, summarized in Table 2, showed that empty liposomes were small (~100 nm). These liposomes were also characterized by slight inhomogeneity (PI 0.36) and negative zeta potential (−16 mV), due to the charge carried by P90G. Quite similar results were obtained for empty PG-PEVs (Table 2). It is worth noting that, for both liposomes and PEVs, the incorporation of the extract induced an increase in size (115 nm, *p* < 0.05; Table 2), with a significant improvement of the homogeneity of the systems, as the polydispersity index values were around 0.27 (*p* < 0.05; Table 2). No relevant variations of the zeta potential values were detected (*p* > 0.05; Table 2).

The entrapment efficiency of the vesicles was calculated based on the content of capsaicin, one of the most abundant components of the white *Capsicum* extract, widely responsible for the pungency of the pepper fruits [53]. The entrapment efficiency was >85% for both vesicle systems (*p* > 0.05; Table 2), which points to their great capability of loading a complex, multicomponent plant extract, such as the white *Capsicum* extract. These values were higher than those obtained in other studies available in the literature, which investigated the encapsulation of black pepper extract [54].

**Figure 1:**
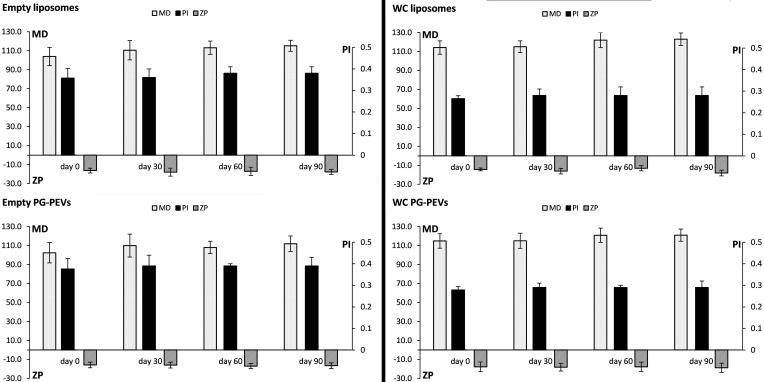
Long-term stability of the vesicle formulations assessed by monitoring mean diameter (MD), polydispersity index (PI), and zeta potential (ZP) for 90 days.

The stability of the white *Capsicum* formulations was evaluated for three months by monitoring the mean diameter, PI, and zeta potential of the vesicles. The results showed no statistically significant variations of the three examined parameters (*p* > 0.05; Figure 1).

### 3.2. Effect of WC Nanoincorporation in Phospholipid Vesicles on Cell Viability

**Figure 2:**
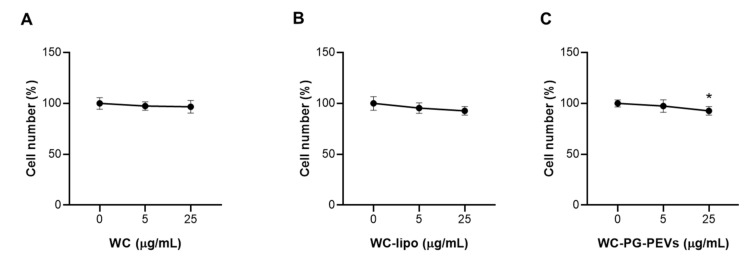
Effect of free WC (**A**), WC liposomes (**B**), and WC PG-PEVs (**C**) on cell viability. U937 cells were counted after 48 h exposure to WC, WC liposomes, and WC PG-PEVs (5 and 25 µg/mL). Means ± S.D. of three replicate independent experiments are shown. Statistical significance of differences was determined by one-way ANOVA followed by Dunnett’s test; * *p* < 0.05. Abbreviations: WC, free WC; and WC-lipo, WC liposomes.

The effect of the white *Capsicum* extract, either free or incorporated in liposomes and PG-PEVs, was assessed in cells. The exposure to free WC for 48 h did not affect cell viability, as no differences from the control (i.e., untreated cells = 0 WC) were detected at the two tested concentrations (Figure 2A).

The incorporation of the extract in both liposomes and PG-PEVS had no effect on the number of viable cells (Figure 2B,C). In particular, WC liposomes did not affect cell viability at both tested concentrations in a significant manner (Figure 2B), while at 25 µg/mL, WC PG-PEVs decreased the cell number by about 10% with respect to untreated cells (Figure 2C), but they cannot be considered toxic. Thus, equivalent doses of free and vesicular WC in both liposomes and PG-PEVs were safe for U937 cells.

### 3.3. Effect of WC Nanoincorporation in Phospholipid Vesicles on ROS Production

**Figure 3:**
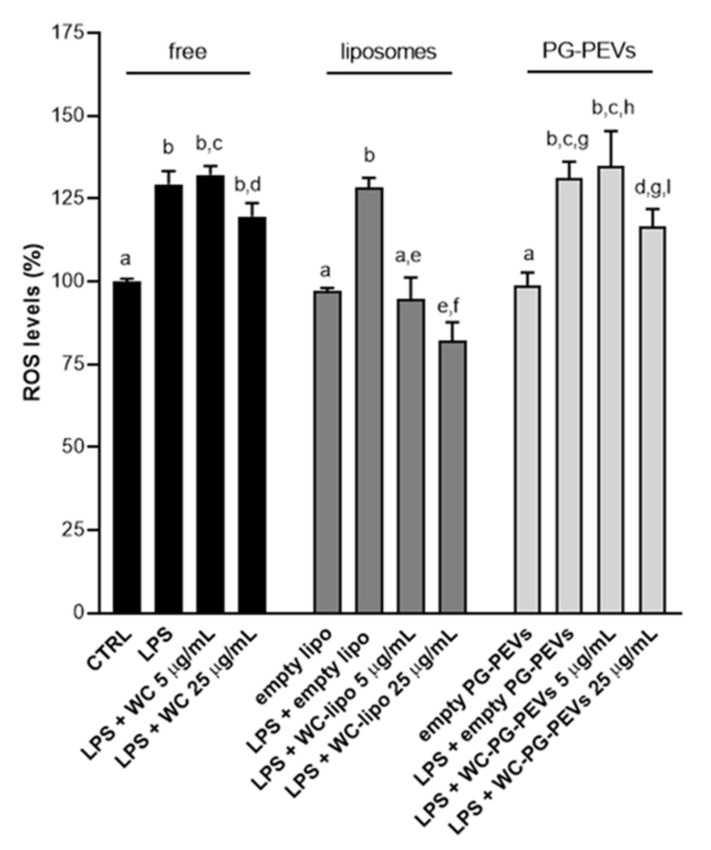
Effect of free WC, WC liposomes, and WC PG-PEVs on ROS production. U937/PMA cells were activated by LPS in both the presence and absence of WC, WC liposomes, and WC PG-PEVs (5 and 25 µg/mL). After 24 h, ROS levels were quantified. Means ± S.D. of four replicate independent experiments are shown. According to one-way ANOVA, differences were significant (*p* < 0.05). Thus, Tukey’s test for multiple comparisons was performed and different letters above the bars indicate significant differences between treatments at *p* < 0.05. Abbreviations: CTRL, untreated cells; WC, free WC; empty lipo, empty liposomes; and WC-lipo, WC liposomes.

In LPS-triggered U937/PMA cells, the white *Capsicum* extract reduced intracellular ROS levels of about 8% only at the highest tested concentration (25 µg/mL) (Figure 3) [45]. No effect on ROS production was observed when cells were treated with the lower concentration (5 µg/mL), as ROS levels were approximately the same as in cells activated with LPS (Figure 3) [45]. The aim of this study was to exploit phospholipid vesicles to enhance the bioavailability of the bioactive compounds present in the white *Capsicum* extract and to facilitate the entry into cells in order to increase the biological response, specifically the anti-inflammatory activity. To this end, we evaluated the effect of WC liposomes and WC PG-PEVs on ROS production induced by LPS in U937/PMA cells. As shown in Figure 3, empty liposomes and empty PG-PEVs did not affect ROS levels after 24 h treatment, in both the presence and absence of LPS. ROS levels increased only when LPS was added, with values around 30% in all the sets of experiments (Figure 3). When WC was incorporated in PG-PEVs, a reduction of about 20% in ROS levels was recorded in cells treated with 25 µg/mL of WC PG-PEVs; 5 µg/mL of WC PG-PEVs was not enough to influence ROS production induced by LPS.

When the cells were treated with WC liposomes, a dose-dependent reduction and a re-establishment of physiological conditions were observed (Figure 3). In more detail, in the presence of 5 µg/mL of WC liposomes, ROS levels were the same as in both unstimulated (CTRL) and empty liposomes-treated cells, which means that the effect of LPS was abolished (Figure 3). Furthermore, at the higher tested concentration (25 µg/mL), WC liposomes reduced ROS levels below physiological values (Figure 3). Hence, liposomal formulation was proved to be the best delivery system for WC to control inflammation induced by LPS by hindering ROS production.

### 3.4. Effect of WC Nanoincorporation in Phospholipid Vesicles on NO Levels

**Figure 4:**
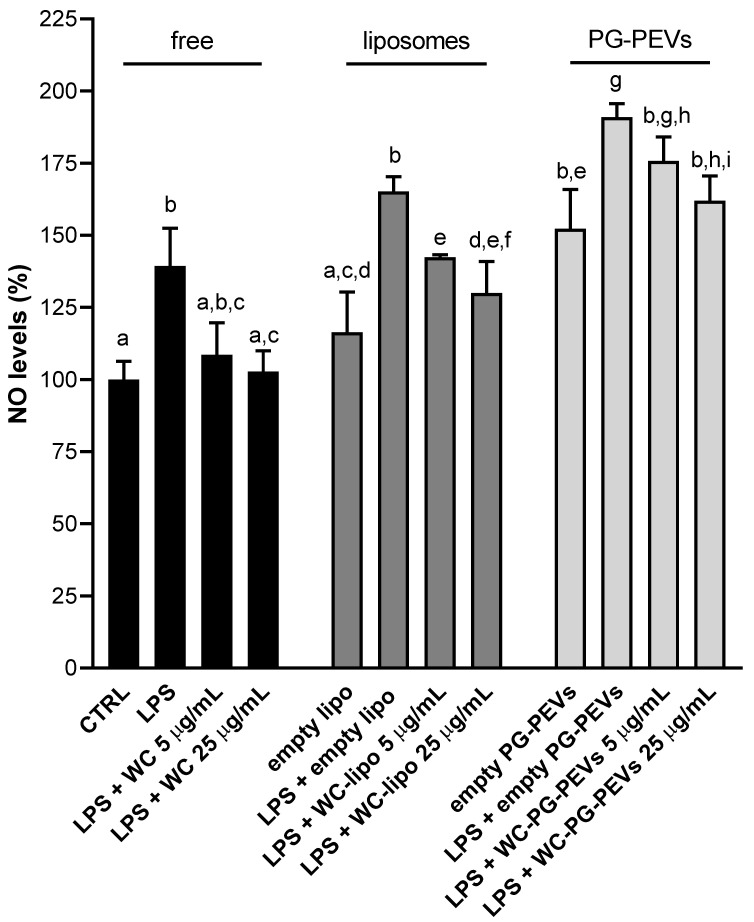
Effect of free WC, WC liposomes, and WC PG-PEVs on NO production. LPS-triggered U937/PMA cells were treated with 5 and 25 µg/mL of WC, WC liposomes, and WC PG-PEVs. After 24 h, NO levels were quantified. Means ± S.D. of four replicate independent experiments are shown. Since differences were statistically significant (one-way ANOVA, *p* < 0.05), Tukey’s test was performed and different letters above the bars indicate significant differences between treatments at *p* < 0.05. Abbreviations: CTRL, untreated cells; WC, free WC; empty lipo, empty liposomes; and WC-lipo, WC liposomes.

In consideration of the remarkable ability of WC to reduce ROS levels, we also evaluated the effect of free WC, WC liposomes, and WC PG-PEVs on NO, which has a relevant role in the onset and progression of inflammation. As shown in Figure 4, WC, both in the free and vesicular forms, was able to reduce NO levels in a dose-dependent manner, but not always in a significant way. In particular, free WC restored the physiological conditions, since NO levels were lower than those of LPS-triggered cells (Figure 4). However, even if 5 µg/mL of free WC reduced NO levels of about 20%, differences between “LPS” and “LPS + WC 5 µg/mL” were not statistically significant according to Tukey’s post hoc test (Figure 4). A significant reduction was observed at the higher tested concentration (LPS + WC 25 µg/mL; Figure 4) in comparison to LPS-activated cells. Both empty liposomes and PG-PEVs induced NO release, even if differences between “CTRL” and “empty lipo” were not statistically significant (Figure 4). Empty PG-PEVs had a more marked effect on rising NO levels (Figure 4). WC liposomes decreased NO production in a significant manner of about 15 and 20% at 5 and 25 µg/mL, respectively, in comparison to LPS-stimulated cells. The treatment with 25 µg/mL of WC PG-PEVs significantly inhibited LPS-induced NO increase by approximately 15% with respect to cells treated with LPS only (Figure 4). The decrease in NO levels induced by 5 µg/mL of WC PG-PEVs was around 8%, but not significant (Figure 4). Overall findings suggested that WC incorporated in liposomes had a stronger ability to reduce NO production than PG-PEVs.

### 3.5. Intracellular Accumulation of White Capsicum Extract

**Figure 5:**
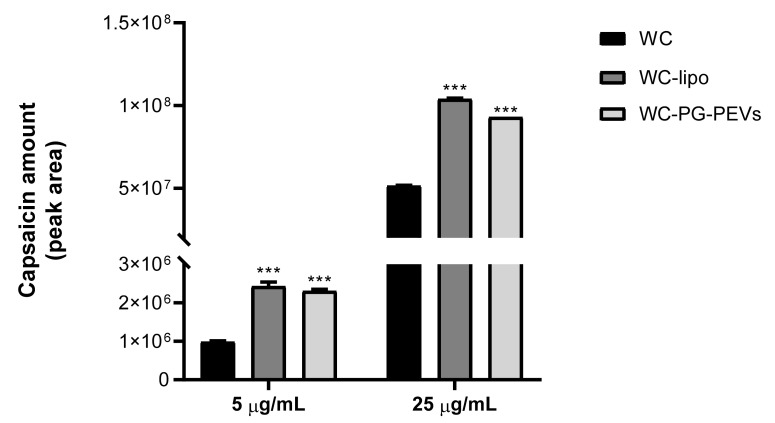
Amount of white *Capsicum* extract, reported as an area of the chromatographic peak of capsaicin, accumulated in U937/PMA cells as free or incorporated into liposomes and PG-PEVs. Two different concentrations of the extract were tested (5 µg/mL and 25 µg/mL). Results are reported as means of three independent replicates ± S.D. (*n* = 3). Statistical significance of differences was determined by using Student’s *t*-test, *** *p* < 0.001 (5 µg/mL WC-lipo vs. 5 µg/mL WC, 5 µg/mL WC PG-PEVs vs. 5 µg/mL WC, 25 µg/mL WC-lipo vs. 25 µg/mL WC, and 25 µg/mL WC PG-PEVs vs. 25 µg/mL WC). Abbreviations: WC, free WC; and WC-lipo, WC liposomes.

The intracellular uptake of the white *Capsicum* extract delivered by the phospholipid vesicles was assessed and compared with the uptake values obtained after incubation of cells with the free extract. LC-MS/MS analyses were carried out and capsaicin was used as marker compound for relative quantification purposes. Two different concentrations of the extract were tested, 5 µg/mL and 25 µg/mL. The results obtained clearly showed that the intracellular uptake of the extract was higher when it was formulated in liposomes and PG-PEVs (Figure 5). Indeed, the amount of capsaicin detected within the cells increased (~2 fold) when the extract was incorporated into the vesicles, rather than in solution, at the two tested concentrations. Among the investigated formulations, slightly higher values of intracellular accumulation were obtained for WC liposomes (~2 and 2.5 fold vs. free extract) compared to WC PG-PEVs (~1.8 and 2.3 fold vs. free extract).

It is worth noting that an approximate 50-fold increase in capsaicin content was detected in cells treated with the higher WC concentration with respect to the lower concentration (25 vs. 5 µg/mL), which did not correspond to the only 5-fold more concentrated extract samples. This could be due to a matrix effect, which gave rise to a higher suppression of the capsaicin signal within the less concentrated sample (5 µg/mL) [55].

These results indicate that liposomes were more efficient than PG-PEVs in transporting the white *Capsicum* extract into the cells and promoting the accumulation of its components into the cytoplasm. This might explain the superior anti-inflammatory activity of WC liposomes, as shown by ROS and NO inhibition studies.

## 4. Discussion

*Capsicum* peppers are recognized as a source of capsaicinoids, phenolic compounds, and antioxidants. Epidemiological studies have shown the benefits of pepper consumption in reducing mortality and improving the quality of health [56]. Many nutraceutical benefits are associated with the consumption of chili peppers, including anti-inflammatory [57], analgesic [58], blood glucose regulation [59], and antioxidant benefits [60,61]. *Capsicum* peppers are frequently attributed functional properties mainly because these foods are sources of carotenoids, vitamin C, vitamin E, alkaloids, flavonoids, and capsaicinoids, which are the predominant phenolic compounds [56]. However, the levels of these bioactive compounds can vary in relation to various factors, such as genotype, stage of maturation, and conditions of growth and harvest [56].

In the present work, the in vitro efficacy of different formulations (free form and vesicle-based) of a methanolic extract of a new pepper genotype in regulating inflammatory responses was evaluated. Liposomal encapsulation strategy has been demonstrated in various studies to improve the therapeutic indices and pharmacological activities of conventional drug formulations [41,62]. In addition, the use of lipid-based nanoparticles has become increasingly popular due to their ability to alter the biopharmacological properties of entrapped hydrophobic drugs (e.g., by improving drug solubility, dissolution kinetics, and bioavailability) [63,64,65].

We investigated the ability of the white *Capsicum* extract to inhibit ROS and NO inflammatory mediators using a sensitive LPS-stimulated macrophage model. ROS and NO are two radical species with well-defined roles in the onset and progression of the inflammatory process. ROS, both as byproducts of numerous enzymatic reactions in different cell compartments, and as generated by specific enzymes such as NADPH oxidase, mediate both physiological and pathological signal transduction [66,67]. NO is a molecule used as a signaling or toxic agent between cells. It plays physiological roles in mammals, acting as a vascular relaxant, neurotransmitter, and inhibitor of platelet aggregation. NO is also generated during immune and inflammatory responses [68]. Therefore, as inflammatory mediators, ROS and NO are important targets in the treatment of inflammatory diseases.

In a previous work [45], we evaluated the anti-inflammatory activity of raw white *Capsicum* extract, the results showing that WC was not toxic to U937 cells at 5 and 25 µg/mL concentrations. In the present study, similar results of non-toxicity were obtained when the cells were treated with WC incorporated into phospholipid vesicles (Figure 2).

Furthermore, liposome incorporation strongly improved the ability of WC to dampen ROS and NO levels in a dose-dependent fashion. Indeed, we observed a good efficiency in bringing ROS back to levels of unstimulated cells, already at the lower tested concentration (5 µg/mL) and an even more marked efficacy was detected at the higher tested concentration of WC (25 µg/mL) (Figure 3). In addition, we found that 5 µg/mL of WC liposomes reduced NO levels in LPS-activated U937/PMA cells in a similar percentage to free WC, but in a statistically significant way. At the concentration of 25 µg/mL, the reduction was more marked, so as to bring NO levels back almost to physiological levels (Figure 4). On the other hand, the incorporation into PG-PEVs did not significantly strengthen the anti-inflammatory proprieties of WC.

The delivery of WC by liposomes was found to facilitate the transport of the extract components across the cell membrane and their accumulation into the cytoplasm, as demonstrated by a double amount of capsaicin detected within the cells after the exposure to WC liposomes in comparison with WC solution, and slightly higher than that obtained with PG-PEVs (Figure 5). This was reflected in a dose-dependent reduction mostly in ROS levels stimulated by LPS and a re-establishment of physiological conditions.

## 5. Conclusions

To the best of our knowledge, this is the first study that reports the incorporation of the new white *Capsicum* genotype extract into phospholipid vesicles and the assessment of its anti-inflammatory activity in cells. The results showed the potential of liposomes in facilitating the entrance of the extract components into cells and the consequent counteraction of ROS and NO deleterious intracellular damage. Further research will be needed to investigate the mechanisms through which white *Capsicum* extract loaded liposomes work, with the identification of the molecular pathways involved in the observed anti-inflammatory activity.

## Figures and Tables

**Table 1:** Composition of vesicle formulations.

Formulation	P90G ^1^	PG ^2^	WC ^3^ Extract	H_2_O
Empty liposomes	120 mg			1 mL
WC liposomes	120 mg		2 mg	1 mL
Empty PG-PEVs	120 mg	0.05 mL		0.95 mL
WC PG-PEVs	120 mg	0.05 mL	2 mg	1 mL

^1^ P90G, phospholipid; ^2^ PG, propylene glycol; ^3^ WC, white *Capsicum*.

**Table 2:** Characteristics of empty and white *Capsicum* (WC) loaded vesicles: mean diameter (MD), polydispersity index (PI), zeta potential (ZP), and entrapment efficiency (E). Each value represents the mean ± S.D. (*n* > 10). *, ° values statistically different (*p* < 0.05) from empty liposomes and empty PG-PEVs, respectively.

Formulation	MD nm ± SD	PI ± SD	ZP mV ± SD	E % ± SD
Empty liposomes	104 ± 9.6	0.36 ± 0.04	−16 ± 2.5	--
WC liposomes	* 114 ± 7.1	* 0.27 ± 0.01	−14 ± 1.5	Capsaicin 86 ± 6.0
Empty PG-PEVs	102 ± 10.7	0.38 ± 0.05	−16 ± 3.0	--
WC PG-PEVs	° 115 ± 7.6	° 0.28 ± 0.02	−18 ± 5.1	Capsaicin 89 ± 2.2

## Data Availability

The data presented in this study are available in article.
